# Fast One‐Step Fabrication of Highly Regular Microscrolls with Controllable Surface Morphology

**DOI:** 10.1002/advs.202302103

**Published:** 2023-05-10

**Authors:** Achim M. Diem, Joachim Bill, Zaklina Burghard

**Affiliations:** ^1^ Institute for Materials Science University of Stuttgart 70569 Stuttgart Germany

**Keywords:** 3D microstructures, footprint, graphene oxide, microscrolls, nanocellulose, rolled‐up nanotechnology, rolling, vanadium pentoxide

## Abstract

Although rolling origami technology has provided convenient access to three‐dimensional (3D) microstructure systems, the high yield and scalable construction of complex rolling structures with well‐defined geometry without impeding functionality has remained challenging. The straightforward, one‐step fabrication that uses external mechanical stress to scroll micrometer thick, flexible planar films with centimeter lateral dimensions into tubular or spiral geometry within a few seconds is demonstrated. The method allows controlling the scrolls’ diameter, number of windings and nanostructured surface morphology, and is applicable to a wide range of functional materials. The obtained 3D structures are highly promising for various applications including sensors, actuators, microrobotics, as well as energy storage and electronic devices.

## Graphical abstract

Mechanically induced, high‐speed scrolling of thin films yields 3D microstructures in a sustainable manner. The method does not require any additional components or treatments and allows controlling the number of windings as well as tailoring the surface morphology of the rolled‐up structures. It furthermore provides access to extended, regular scroll arrays that are promising for multitask applications.

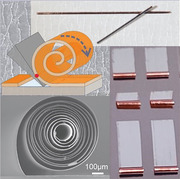

## 1 Introduction

Progress in micro‐/nanotechnology has been driven by the need to construct three‐dimensional (3D) micro‐/nanostructures with tailored geometry and functionality^[^ ^1^, ^2^ ^]^ In this manner, the material's footprint can be significantly reduced, while simultaneously preserving its surface area, which is highly attractive for applications like smart systems,^[^ ^3^, ^4^ ^]^ as well as components for energy storage,^[^ ^5^ ^]^ electronic,^[^ ^6^ ^]^ and optical devices.^[^ ^7^ ^]^ One particularly promising strategy is rolling origami, where planar films or nanomembranes are rolled‐up into a wide variety of 3D shapes with intriguing structure design ability.^[^ ^3^ ^]^ This approach critically depends on implementing a vertical strain gradient into the flexible layers, which has mainly been achieved through a lattice mismatch between the two layers comprising the planar film.^[^ ^8^ ^]^ The strain is released through controlled removal of an underlying sacrificial layer, via chemical etching^[^ ^9^ ^]^ or dissolving.^[^ ^10^ ^]^ However, the etching conditions required to remove the sacrificial layer limit the material choice. An alternative option is to induce spontaneous delamination of the film, for instance through thermal stress,^[^ ^11^ ^]^ liquid intercalation,^[^ ^12^ ^]^ or preferred swelling of one of the layers.^[^ ^13^ ^]^

Though valuable, these approaches still suffer from drawbacks hindering their large‐scale application.^[^ ^8^ ^]^ First, the maximum film area that can be rolled‐up is restricted to the µm^2^ range, leading to only a certain number of windings. Although some progress has recently been made by using a magnetic field to guide the rolling direction, a sacrificial layer is still required.^[^ ^14^, ^15^ ^]^ Second, they are quite time consuming, with a typical roll‐up speed on the order of hundreds of nanometers per second.^[^ ^8^, ^16^ ^]^ Accordingly, rolling‐up larger areas (cm^2^ range) requires > 10 h.^[^ ^5^ ^]^ Furthermore, due the sizable number of involved steps and material components, these methods suffer from a high energy consumption and environmental footprint. Hence, there is a strong need for alternative, eco‐friendly roll‐up methods, which allow for fast rolling over large dimensions, efficient control over the scroll diameter and winding number, as well as a high fabrication throughput and easy up‐scaling.

## 2 Results and Discussion

Here, we demonstrate highly regular microscrolls with controllable surface morphology fabricated in a single step, wherein a strain gradient is introduced into a thin, flexible film via application of external mechanical stress. Remarkably, the mechanically‐induced scrolling is feasible over several centimeter in any desired lateral direction, yielding scrolls with diameters up to several hundreds of micrometers, an aspect ratio exceeding 600, and an associated foot‐print reduction by a factor of up to 45. Moreover, the unprecedented scrolling speed of 1 mm s^−1^ enables scroll fabrication within a few seconds, more than one order of magnitude faster than for state‐of‐the‐art techniques.^[^ ^2^, ^3^, ^8^ ^]^ While these capabilities are exemplified for vanadium pentoxide (V_2_O_5_) thin films, which are promising, for example, applications in energy storage devices,^[^ ^17^, ^18^ ^]^ the method is applicable to a wider range of materials. The investigated V_2_O_5_ films with a thickness ranging between 500 nm and 5 µm are self‐assembled from V_2_O_5_ nanofibers onto a Si/SiO_2_ substrate (Figure S1, Supporting Information). The fabrication and mechanical properties of the films have been previously reported.^[^ ^19^, ^20^ ^]^

**Figure 1:**
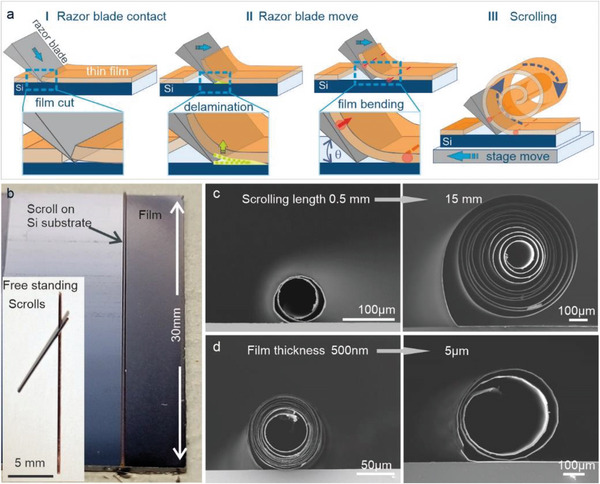
Microscroll fabrication and dimensions. a) Schematic illustration of the scrolling procedure. b) Optical images of a scroll formed from a 2 µm thick V_2_O_5_ film on a Si substrate, and a few free‐standing (inset). c) SEM cross‐sections of scrolls obtained from a 2 µm thick V_2_O_5_ film over 0.5 mm (diameter of 80 µm/two windings) and 15 mm (diameter of 650 µm/12 windings). d) SEM cross‐ sections of scrolls obtained from a V_2_O_5_ film with a thickness of 500 nm and 5 µm, in both cases by scrolling over 3 mm. The thicker film yields a significantly larger scroll diameter and a smaller number of windings (13 vs 3). Scrolling speed was 1 mm s^−1^ and the blade contact angle 45°.

The devised scrolling setup (Figure S2, Supporting Information) consists of a linear motion stage, whose driving speed (on the order of mm s^−1^) and moving direction can be controlled, combined with a static razor blade (edge thickness of 250 nm (Figure S3, Supporting Information). The scrolling procedure, whose individual steps are observable by optical microscopy (Figure S4 and Movie S1, Supporting Information), is schematically outlined in **Figure** **1**a. After mounting the substrate covered by the V_2_O_5_ film on the stage, the razor blade is lowered onto the film's surface (Figure S4 and Movie S2, Supporting Information) under a certain angle (*θ*), and fixed with a weight to ensure a tight contact with the substrate accompanied by a film cut (Movie S2 and Figure S5, Supporting Information). Film scrolling is then accomplished by moving the stage toward the stationary blade, whereupon the V_2_O_5_ film is forced to delaminate and bent under a certain angle (Figure 1a), yielding a scroll that remains attached to the substrate (Figure 1b). The scrolls are mechanically sufficiently stable to be manually detached from the substrate and further manipulated as free‐standing objects (Figure 1b insert).

The appreciable scrolling length provides access to scrolls with a large winding number, and an outer diameter of several tens to several hundreds of micrometers (Figure 1c), while their length of up to 30 mm is determined by the blade and substrate size. Through a suitable design of the razor blade edge, it becomes possible to fabricate entire arrays of scrolls at well‐defined positions in a single step (Figure S6a, Supporting Information). Furthermore, by moving a razor blade diagonally across the substrate or by cutting the film in diagonal stripes, a spring‐like geometry with defined shape and size can be obtained (Figure S6b, Supporting Information). As a further observation, thinner films yield scrolls of smaller diameter (i.e., more windings) for the same scrolling length, as compared to thicker films (Figure 1d). This trend is in accord with the increase of flexural rigidity with film thickness.^[^ ^21^ ^]^ Furthermore, thinner films allow for a larger footprint reduction as well as aspect ratio (Figure S7, Supporting Information).

**Figure 2:**
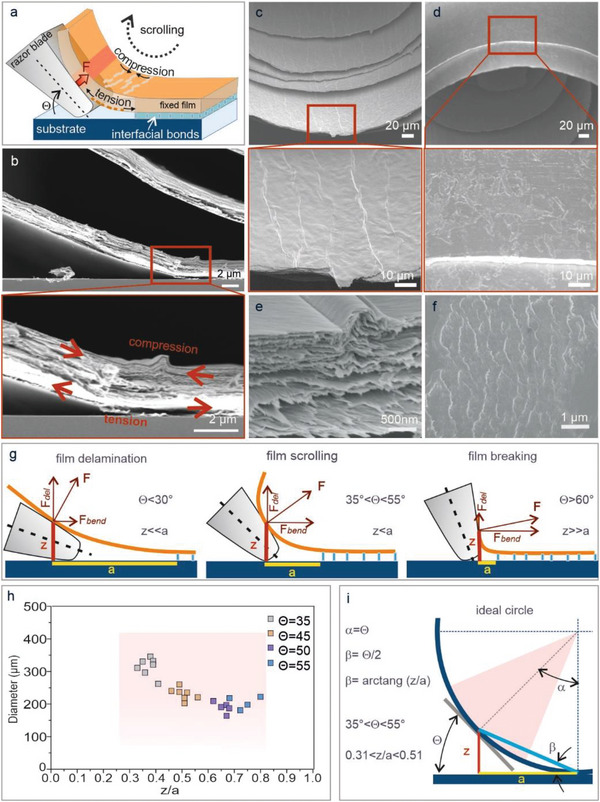
Scrolling mechanism and microscroll morphology. a) Schematic illustration of the mechanically‐induced film scrolling. The razor blade with trapezoid shape acts like a wedge to delaminate and bent the film due to the force *F* induced by moving the film toward the razor blade. This introduces a vertical strain gradient, accompanied by a compression (tension) of the inner (outer) surface of the bent film. b) SEM cross‐section of the bent film layer. c,d) SEM cross‐sections of a scroll obtained from a 2 µm thick V_2_O_5_ film, showing the inner (c) and outer (d) surface. e) SEM image of a representative fold on the inner surface layer. f) SEM images of the outer surface of the layer, revealing breaking of the layer due to tension stress. g) Schematic representation of the relevant geometric parameters of the scrolling process, where *a* is the length, z is the vertical deflection of the delaminated film, and *F* the force with its delamination (*F*_del_) and bending (*F*_bend_) components, shown for three contact angles (*θ*) of the razor blade. h) Plot of scroll diameter versus z/a ratio for four contact angles of the blade. i) Sketch of the film curvature assuming an ideal circle. The tangent corresponds to the razor blade surface. The red triangle indicates the contact angle range over which scroll formation is feasible.

Microscopic inspection of the scroll formation and their microstructure revealed two major processes that occur simultaneously, namely film delamination and bending (**Figure** **2**a). Such delamination is well known from asymmetric wedge tests used to investigate the fracture energy of adhesive joints.^[^ ^22^ ^]^ However, as a major difference to a wedge with its flat end, the razor blade edge has a trapezoid shape, which facilitates bending of the delaminated film into circular shape due to the vertical strain gradient introduced in the contact zone (indicated by the red region in Figure 2a). This bending is analogous to that of a single cantilever beam, with the inner region experiencing compression, while the outer one is subjected to tension stresses.^[^ ^23^ ^]^

In accord with established theories for wedge tests^[^ ^22^ ^]^ and single cantilever beam adhesion tests,^[^ ^23^ ^]^ besides scrolling length (Figure 1c) and film thickness (Figure 1d), the contact angle of the razor blade, the blade sharpness, as well as the scrolling speed influence the force acting on the delaminated film and thus represent suitable parameters to control the scrolling process.

Scrolling works for the contact angle range between 30° and 60°. This limited angle range can be explained by the elastic‐plastic deformation of the film due to the applied bending moment,^[^ ^21^, ^23^ ^]^ as schematically illustrated in Figure 2g. The latter is determined by the length *a* of the delaminated film, the deflection *z*, and the force *F* acting perpendicular to the film plane (Figure S8, Supporting Information). This force has two components acting toward delamination (*F*_del_) and bending (*F*_bend_), respectively. For angles below 30° (left panel), the bending force *F*_bend_ is too small to induce plastic deformation of the film required for scrolling, and accordingly delamination of the film and its elastic deformation is dominant (z << a) (Movie S3, Supporting Information). For angles above 60° (right panel), the situation is opposite, that is, film delamination is weak while the bending force is so strong involving plastic deformation such that the film breaks (z << a). By contrast, intermediate angles around 45° (middle panel) allow for a proper balance between delamination and bending and accompanied film's deformations. Notably, the angle dependence of the acting forces can be exploited to control the scroll diameter (Figure S9, Supporting Information). For quantitative evaluation, the scroll diameter is plotted in Figure 2h for different blade angles against the ratio *z*/*a*, with the latter directly reflecting the balance between delamination and bending. In general, scrolling proceeds smoothly when *a* is approximately doubled observed for 45° angle. It should be noted that in the case of very strong interfacial bonding (compared to the bending force), film delamination is limited, such that the film will be scratched from the surface rather than smoothly scrolled. It is apparent that the scroll diameter decreases with increasing scrolling angle, while the *z*/*a* ratio increases from approximately 0.3 to 0.8. Our experimental data are close to the values valuable for a circle (Figure 2i), thus accounting for the observed circular shape of the scrolls. Assuming a tangential contact line between film and blade surface, for 35° the most suitable z/a ratio is calculated to be 0.31, for 45° 0.41, and for 55° 0.51.

**Figure 3:**
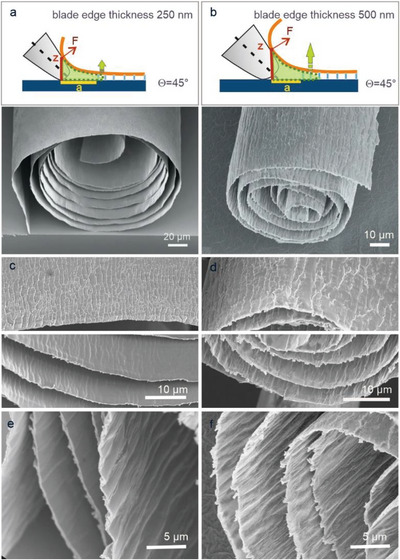
Influence of blade sharpness on microscroll morphology. a,b) Schematic illustration of the relevant scrolling parameters. The delamination region is marked by in green. The corresponding SEM cross‐sections at the bottom belong to scrolls obtained from of a 500 nm thick V_2_O_5_ film scrolled with a 250 (a) and 500 nm (b) thick blade edge, in both cases with a scrolling speed of 1 mm s^−1^ and a contact blade angle of 45°. c,d) Corresponding SEM images of the outer and inner surface of the scrolled film. e,f) Higher magnification SEM images of the interior layers of the scrolls in (c) and (d).

Another relevant finding is that the blade edge thickness allows tuning of the scroll diameter and surface morphology, especially when the film thickness is comparable to the blade edge thickness. As apparent from Figure S3 (Supporting Information), a larger blade edge thickness (500 vs 250 nm) leads under otherwise identical conditions to a significantly smaller inner scroll diameter as small as 10 µm (**Figure** **3**a,b). Furthermore, the outer surface appears rougher due to the formation of regular scales composed of obtruded layers (Figure 3c,d). At the same time, the inner surface displays a wave‐like morphology, although the associated folds are more pronounced for the thicker edge (Figure 3e,f). This difference can be explained by the stronger lifting of the delaminated film in case of the thicker edge (green area in the Figure 3a), which results in stronger film bending and correspondingly more pronounced plastic deformation. The stronger bending/delamination in turn affects the surface morphology and accordingly the effective surface area of the scrolls. These features highlight that the choice of the razor blade and film thickness allows not only to adjust the inner and outer diameter of the scrolls, but also to tailor their surface morphology, which is of interest for applications requiring a high surface area of the material. More importantly, such “shark skin morphology” of the scroll is promising for actuator applications, for example, to facilitate opening and closing of the surface during moving and lifting objects. It should be noted that for films with different mechanical properties, achieving such surface morphology requires adjusting the film thickness and accordingly also the blade radius. Besides the films’ mechanical properties, the surface morphology will be influenced also by the specific shape and size of their building blocks (i.e., fibres, sheets, or platelets). Furthermore, the adhesion strength of the film on the substrate must be considered.

Another useful parameter to tailor the geometry and morphology of the scrolls is the scrolling speed. For the same scrolling angle and film thickness, slower scrolling (0.1 mm s^−1^) causes the film to fully delaminate (Movie S4, Supporting Information), while faster scrolling (5 mm s^−1^) results in skipping over the film surface. A speed of 1 mm s^−1^ provided best results, although a systematic investigation of the impact of the scrolling speed remains to be done. Nonetheless, this impressive scrolling speed (compared to 0.5^[^ ^16^ ^]^ or 5 µm min^−1[^ ^11^ ^]^ reached previously) renders this approach promising for large scale fabrication.

**Figure 4:**
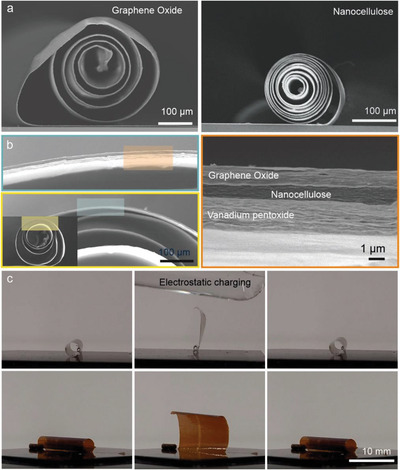
Scrolling of other types of film. a) SEM cross‐sections of scrolls obtained from 2.3 µm thick graphene oxide and 2.5 µm thick nanocellulose films. The scrolling length was 3 mm, and the speed 1 mm s^−1^. b) SEM cross‐section of a scroll obtained from a 4 µm thick (GO/NC/V_2_O_5_) hetero‐layered film, testifying the possibility to combine different materials as electrodes and separators toward electrochemical energy storage applications. The razor blade angle was 45°, the scrolling length 10 mm. The diameter of the hybrid scroll is 1 mm. c) Optical images of electrostatic charging induced opening and closing of a V_2_O_5_ scroll, fabricated from a 2 µm thick film (area 10 mm x 10 mm).

The developed scrolling method can be readily extended to other types of films and combinations thereof, opening up versatile applications in various fields such as energy storage, actuators or micro robotics.^[^ ^8^ ^]^ We demonstrate scrolled graphene oxide (GO) and nanocellulose (NC) films (**Figure** **4**a), which are obtainable through a similar self‐assembly approach like the V_2_O_5_ films and display different mechanical properties.^[^ ^24^, ^25^ ^]^ As the layered structure of GO films, involving GO sheets, renders them stiffer than NC films being composed of entangled nanofibers, the former yield larger diameter scrolls, whose windings are less tightly packed in comparison to the NC scrolls.

Our method allows scrolling of a heterotrilayer consisting of GO, V_2_O_5_ and NC, as shown in Figure 4b. The film was fabricated by subsequent deposition of the films, starting with GO, using the procedures described in SI for each layer. The scroll diameter of ∼1 mm is due to the total film thickness of 4 µm, combined with the quite large scrolling length of 10 mm. Microscopic analysis revealed that the three films remain closely attached to each other, without any apparent damage on the outer and inner scroll surface. As the individual films possess a certain density of —OH groups, and were deposited under similar conditions (self‐assembly from aqueous dispersions), we assume that they are connected via weak hydrogen bonds, which are easily broken and restored again, and accordingly the overall structural integrity is preserved.^[^ ^19^, ^20^ ^]^ Thus, it is important to ensure an appropriate adhesion strength between the films, in order to achieve scrolling without film separation. It is noteworthy that there are further parameters that might influence the stress distribution between the layers and thereby also the geometrical structure of the scroll as well as its surface morphology. These include the arrangement of the films based on their mechanical properties, the number of layers composing the film, and the thickness ratio between them. The structural integrity of the hetero‐layered scrolls makes them especially attractive as components of solid‐state batteries wherein both electrodes and solid electrolyte are combined in a compact manner in order to ensure an effective ion/electron exchange.^[^ ^26^ ^]^

Another valuable property of the V_2_O_5_ scrolls with dimensions of up 10 mm x 10 mm is their ability to be repeatedly (speed less than a second) opened and closed by an external electrostatic field, without any detectable damage, as depicted in Figure 4c (see also Movie S5, Supporting Information). The possibility of such electrically‐controlled actuation on the sub‐second timescale testifies that the local plastic deformation introduced during scrolling does not impede the scrolls’ mechanical flexibility. Moreover, it opens up a range of applications where the movement of a large surface area is required, which may be exploited, for example, actuators with muscle‐like performance.^[^ ^27^, ^28^ ^]^

## 3 Conclusions

In conclusion, the demonstrated fast, one‐step method provides access to rolled‐up 3D structures like tubules or scrolls, whose diameter and number of windings can be tuned by the film thickness, scrolling length, and the contact angle of the razor blade. It is principally applicable to almost any type of thin film by ensuring a proper balance between film bending and delamination. This task is feasible through substrate surface modification combined with optimizing the razor blade sharpness and scrolling speed. Moreover, scrolls of well‐defined structural geometry and surface morphology may be achievable through suitable substrate pattering prior to film deposition, combined with a defined scrolling direction. As another promising perspective, it not only allows for scrolling of hetero‐layered films, but could furthermore be extended toward nested scroll architectures, potentially opening access to complex, high performance systems.

## Conflict of Interest

The authors declare no conflict of interest.

## Supporting information

Supporting Information

Supplemental Movie 1

Supplemental Movie 2

Supplemental Movie 3

Supplemental Movie 4

Supplemental Movie 5

## Data Availability

The data that support the findings of this study are available in the supplementary material of this article.
