# Supplementary material for: Fast One‐Step Fabrication of Highly Regular Microscrolls with Controllable Surface Morphology
Source: Adv Sci (Weinh). 2023 May 10;10(21):2302103. doi: 10.1002/advs.202302103 (PMC10375128; doi:10.1002/advs.202302103)
Supplement: Supplementary file 1 — Supporting Information [file ADVS-10-2302103-s006.pdf]

ADVANCED SCIENCE Open Access

Supporting Information

for *Adv. Sci.*, DOI 10.1002/advs.202302103

Fast One-Step Fabrication of Highly Regular Microscrolls with Controllable Surface Morphology

*Achim M. Diem, Joachim Bill and Zaklina Burghard**

Supporting information

Fast one-step fabrication of highly regular microscrolls with controllable surface morphology

Achim M. Diem, Joachim Bill, Zaklina Burghard *

Institute for Materials Science, University of Stuttgart, 70569 Stuttgart, Germany.

*Corresponding author. Email: zaklina.burghard@imw.uni-stuttgart.de.

Materials and Methods

V_2O_5 nanofiber synthesis

A V_2O_5 nanofiber dispersion was prepared from ammonium meta-vanadate (1 g, Fluka) and acidic ion-exchange resin (10 g, Dowex 50WX8, Alfa Aesar) in deionized water. To achieve concentrations of 3.5 mg/mL and 7.6 mg/mL, the volume of used water was 200 mL and 100 mL respectively. Tis mixture was heated to 80 °C for 10 min, cooled to room temperature and then stored for 28 days under ambient conditions, and finally the dispersion was decanted from the ion exchanger. Thus obtained dispersion contains V_2O_5 nanofibers with a length of up to five µm.[1,2]

V_2O_5 thin film fabrication

Details of the fabrication steps have been previously reported.[1] Briefly, the V_2O_5 nanofiber dispersion was poured on Si(100) p-type wafer (Wacker, Sitronic) inside a glass beaker. Evaporation of the water at ambient conditions induced self-assembly of the nanofibers into a micrometer thick film on the substrate. By using different amounts of V_2O_5 fiber solutions, it is possible to control the thickness of the resulting film in steps of approximately 0.2 µm. After complete evaporation of the water, the V_2O_5 thin film was used for scrolling. To obtain V_2O_5 film thicknesses of 500 nm, the dispersion with a concentration of 3.5 mg/mL was diluted with water in a ratio of 1:3. For 5 µm thick V_2O_5 films, the as-prepared dispersion with a concentration of 7.6 mg/mL was directly used.

Graphene oxide (GO) thin film fabrication

The fabrication is detailed in our previous publication.[3] The GO dispersion was purchased from Nanografi (Ankara, Turkey) with a GO concentration of 8 mg/mL and a GO sheet size of 1 to 5 µm an a thickness of 0.4 to 1.1nm. To obtain GO thin films with a thickness of 2.5 µm, the GO dispersion was diluted to 1.61 mg/mL and poured into a polyethylene beaker (whose size leads to 0.46 mL/cm^2) equipped with a cleaned Si(100) p-type wafer (Wacker, Sitronic). After complete drying, the GO thin film was used for scrolling.

Cellulose nanofiber (CNFs) synthesis

CNFs were prepared as described elsewhere[4] from bleached eucalypt (Eucalyptus globulus) kraft pulp. Briefly, an aqueous suspension of the pulp was subjected to a TEMPO-mediated oxidation process. To this end, 2,2,6,6-tetramethylpiperidine-1-oxyl radical (0.016 g, TEMPO), NaBr (0.1 g), and NaClO (10 mmol) were added to the pulp (15 g) and the pH by was adjusted

to 10 adding 1 M NaOH. All reagents were purchased from Sigma-Aldrich. Thus obtained CNF nanofibers exhibit a surface charge of 1546 μmol g−1.

Nanocellulose (NC) thin film synthesis

The CNFs gel was diluted to 1 mg/mL with deionized water and poured onto a cleaned Si(100) p-type wafer (Wacker, Sitronic) inside a polyethylene dish (55 mm diameter). The amount of the mixture was 0.38 ml/cm², yielding 1.5 μm thick CNF thin films.[5]

Hybrid film synthesis

A 4 μm thick heterotrilayer consisting of GO, NC and V_2O_5 films was fabricated by subsequent deposition of the films, starting with GO after complete drying, using the procedures described above for each layer.

Structure characterization

The micro/nanostructure of the films/scrolls was investigated by light microscopy (Keyence Digital Microscope VHX-2000) and scanning electron microscopy (Ultra 55 FEM Microscope Zeiss). Scroll formation was recorded with a Keyence Digital Microscope VHX-2000. Electrostatic charging used for opening the V_2O_5 scrolls was accomplished by rubbing a cotton cloth against the glass rod.

Supplementary Text and Figures

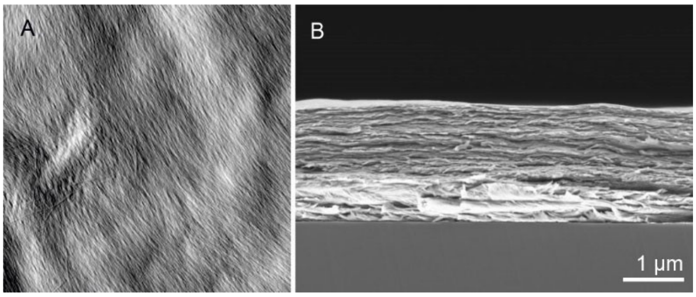

Figure S1. Morphology and microstructure of the V_2O_5 film self-assembled on a Si substrate. (A) AFM image (scan size 1x1 μm) of the film surface revealing well-aligned nanofibers. (B) SEM cross-section of the fracture surface, confirming a uniform layered structure.

After removal from the substrate, the free-standing films with lateral dimensions in the centimeter range and thickness in the micrometer range exhibit extreme flexibility.[1] At the same time, their well-organized nano/microstructure imparts a high yield strength of 120 MPa and Young's modulus of up to 35 GPa.[2]

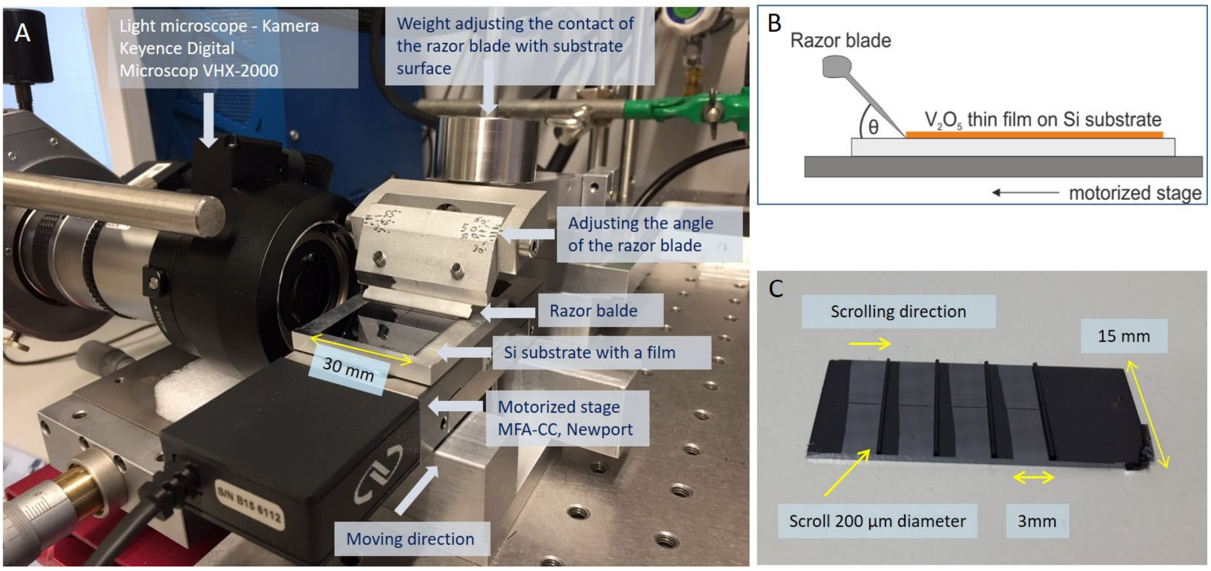

Figure S2. (A) Digital image of the in-house built setup for film scrolling. (B) Schematic depiction of the setup. (C) Digital image of a Si substrate with 4 scrolls obtained from 3 mm (length) x 15 mm (width) areas of a V_2O_5 film with a thickness of 2 µm.

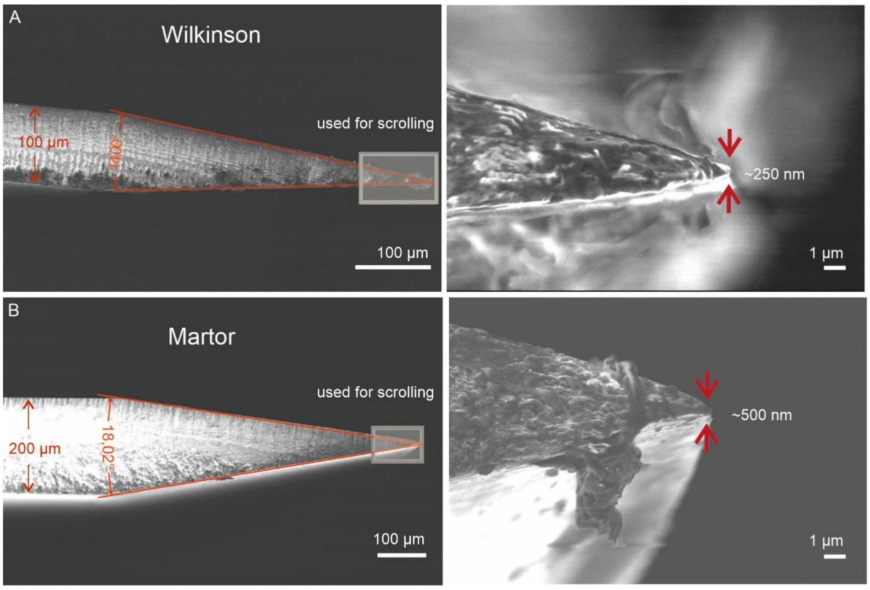

Figure S3. Optical images of cross-sections of the used razor blades with relevant angles and dimensions. (A) Wilkinson classic (Wilkinson Sword GmbH, Germany) blade. (B) Martor (MARTOR KG, Germany) blade. The grey rectangles mark the region involved in the scrolling process.

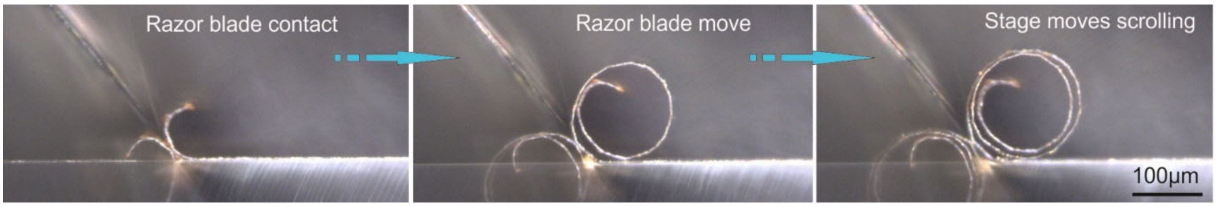

Figure S4. Optical images of the progression of the scrolling process (cross-sections of the formed scrolls). The mirror-like feature to the left of the curved film is an optical reflection.

As the razor blade is manually lowered onto the film's surface under a certain angle (θ), and fixed with a weight (up to 222g), this inevitably causes some lateral slide of the blade, accompanied by the spontaneous formation of a half-scroll with an irregular shape, due to the uncontrolled speed of this initial stage movement (**movie S2**). The geometry of this half-scroll governs the subsequent scrolling, i.e., whether more tube- or spiral-like scrolling occurs (**Figure S5**).

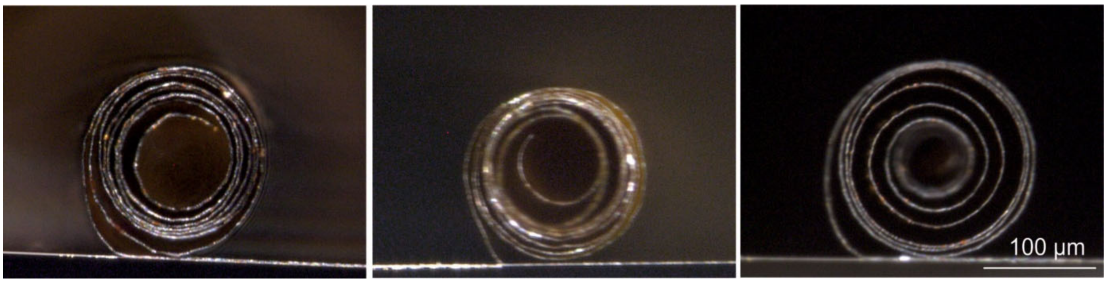

Figure S5. Optical images of three different final scrolls with tube- or spiral-like geometry, depending on the first scroll step. Scrolling length was 3mm.

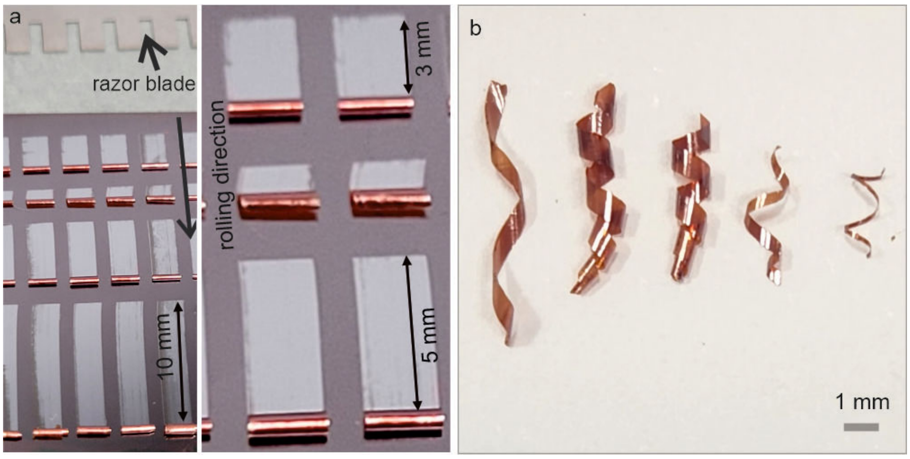

Figure S6. a) Digital images of structured razor blade with a length of 2 mm, used to fabricate an array of micro-scrolls of 2μm thick vanadia film on a Si substrate. b) Digital image of a vanadia film rolled-up into into a helical, spring-like geometry, achieved through cutting the film in diagonal stripes before scrolling.

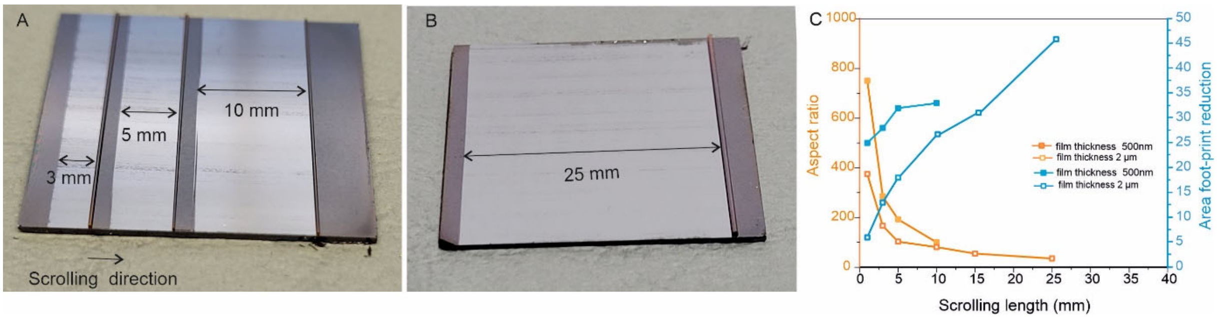

Figure S7. (A and B) Digital image of scrolls formed from a 2µm V_2O_5 film on a Si substrate using different scrolling lengths (3, 5, 10 and 25 mm). (C) Plot of calculated aspect ratio and area foot print reduction of the scrolls as a function of scrolling length, for two films with 500 nm and 2 µm thickness, respectively.

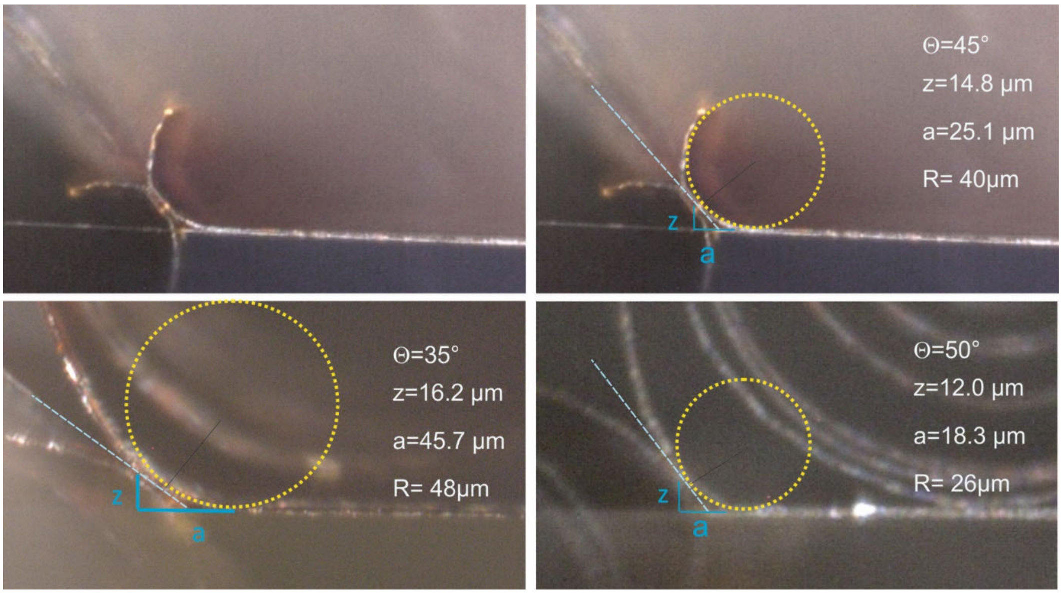

Figure S8. Typical optical images of the formed scrolls, with the relevant parameters (*z*, deflection length, *a*, delamination length and *R*, radius of the bended delaminated film) indicated. These data were used for the evaluation in main text Figure 2h. The mirror-like feature to the left of the curved film is an optical reflection.

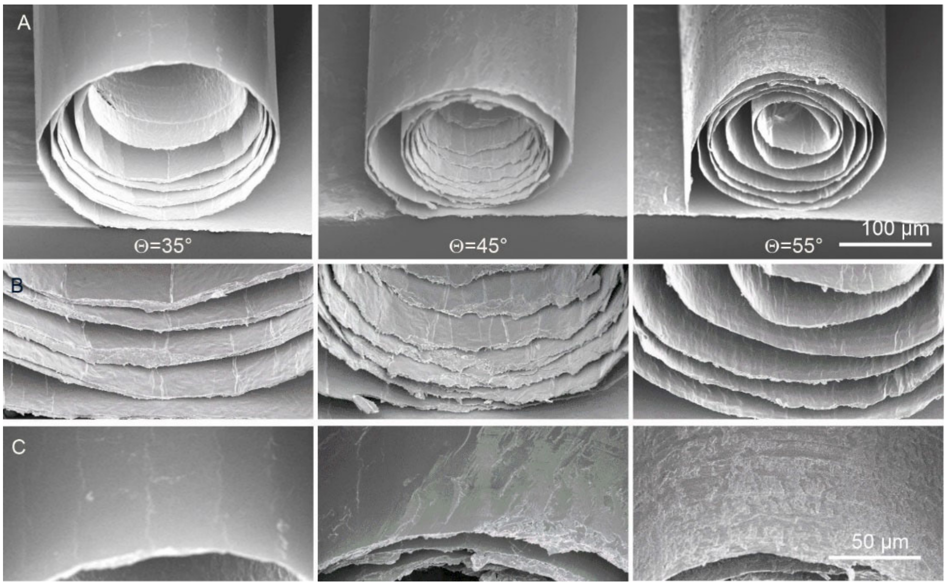

Figure S9. (A) SEM cross-sections of scrolls obtained from a 2 µm thick V_2O_5 film (3 mm scrolling length) for three different contact angles of the blade. (B and C) SEM images of the corresponding inner (B) and outer (C) scroll surfaces.

The angle dependence of the acting forces can be exploited to control the scroll diameter. **Figure S9A** compares the cross-sections of three different scrolls obtained from a 2 µm thick V_2O_5 film for a blade angle of 35°, 45° and 55°, respectively. The observed decrease of scroll diameter with increasing angle is consistent with the corresponding increase of the bending force. The stronger bending manifests itself in more pronounced folds on the significantly compressed inner scroll surface layer (**Figure S9B**), as well as the emergence of broken layers on the outer scroll surface due to the strong tension (**Figure S9C**). The diameter decrease indicates the presence of stronger plastic deformation in the film. Closer inspection of the microstructure of the inner and outer layer (**Figure S9B** and **C**) corroborates this conclusion.

Movie S1. Scroll formation (real time) from a 2 µm thick V_2O_5 film under scrolling angle of 45°, over a length of 3 mm; speed 1 mm/s. real time

Movie S2. Spontaneous formation (real time) of a half-scroll with an irregular shape, which is due to the uncontrolled speed of the initial stage movement.

Movie S3. Scrolling of a 2 µm thick V_2O_5 film (real time) under scrolling angle of 25°, over a length of 3 mm; speed 1 mm/s. Shown is the delamination of the film resulting in one winding with a large diameter of 1 µm.

Movie S4. Scrolling of a 2 µm thick V_2O_y film (real time) using a slower speed of 0.1 mm/s, leading to full delamination of the film.

Movie S5. Electrostatic charging-induced opening and closing of a V_2O_5 scroll
(real time, view 1 and 2).

References

1. Z. Burghard, A. Leineweber, P.A. van Aken, T. Dufaux, M. Burghard, J. Bill, *Adv. Mater.* **2013**, 25, 2468.
2. A. M. Diem, A. Knöller, Z. Burghard, J. Bill, *Nanoscale* **2018**, 10, 15736.
3. A. Knöller, C.P. Lampa, F.V. Cube, T.H. Zeng, D.C. Bell, M.S. Dresselhaus, Z. Burghard, J. Bill, *Sci. Rep.* **2017**, 7, 4099.
4. Ú. Fillat, B. Wicklein, R. Martín-Sampedro, D. Ibarra, E. Ruiz-Hitzky, C. Valencia, A. Sarrión, E. Castro, M.E. Eugenio, *Carbohydr. Polym*. **2018**, 179, 252.
5. B. Wicklein, A.M. Diem, A. Knöller, M.S. Cavalcante, L. Bergström, J. Bill, Z. Burghard, *Adv. Funct. Mater*. **2018**, 28 (27), 1704274.
